# Cortical involvement determines impairment 30 years after a clinically isolated syndrome

**DOI:** 10.1093/brain/awab033

**Published:** 2021-04-21

**Authors:** Lukas Haider, Ferran Prados, Karen Chung, Olivia Goodkin, Baris Kanber, Carole Sudre, Marios Yiannakas, Rebecca S Samson, Stephanie Mangesius, Alan J Thompson, Claudia A M Gandini Wheeler-Kingshott, Olga Ciccarelli, Declan T Chard, Frederik Barkhof

**Affiliations:** 1 NMR Research Unit, Queen Square Multiple Sclerosis Centre, Queen Square Institute of Neurology, University College London, London, UK; 2 Department of Biomedical Imaging and Image Guided Therapy, Medical University Vienna, Austria; 3 Centre for Medical Image Computing (CMIC), Department of Medical Physics and Biomedical Engineering, University College London, London, UK; 4 Universitat Oberta de Catalunya, Barcelona, Spain; 5 Department of Clinical and Experimental Epilepsy, University College London, London, UK; 6 School of Biomedical Engineering and Imaging Sciences, King’s College London, London, UK; 7 Dementia Research Centre, Institute of Neurology, University College London, London, UK; 8 Department of Neuroradiology, Medical University of Innsbruck, Innsbruck, Austria; 9 Neuroimaging Core Facility, Medical University of Innsbruck, Innsbruck, Austria; 10 Department of Brain and Behavioural Sciences, University of Pavia, Pavia, Italy; 11 Brain MRI 3T Research Centre, IRCCS Mondino Foundation, Pavia, Italy; 12 National Institute for Health Research (NIHR) University College London Hospitals (UCLH) Biomedical Research Centre, London, UK; 13 Department of Radiology and Nuclear Medicine, VU University Medical Centre, Amsterdam, The Netherlands

**Keywords:** multiple sclerosis, clinically isolated syndrome, atrophy, magnetic resonance imaging, cortex

## Abstract

Many studies report an overlap of MRI and clinical findings between patients with relapsing-remitting multiple sclerosis (RRMS) and secondary progressive multiple sclerosis (SPMS), which in part is reflective of inclusion of subjects with variable disease duration and short periods of follow-up. To overcome these limitations, we examined the differences between RRMS and SPMS and the relationship between MRI measures and clinical outcomes 30 years after first presentation with clinically isolated syndrome suggestive of multiple sclerosis. Sixty-three patients were studied 30 years after their initial presentation with a clinically isolated syndrome; only 14% received a disease modifying treatment at any time point. Twenty-seven patients developed RRMS, 15 SPMS and 21 experienced no further neurological events; these groups were comparable in terms of age and disease duration. Clinical assessment included the Expanded Disability Status Scale, 9-Hole Peg Test and Timed 25-Foot Walk and the Brief International Cognitive Assessment For Multiple Sclerosis. All subjects underwent a comprehensive MRI protocol at 3 T measuring brain white and grey matter (lesions, volumes and magnetization transfer ratio) and cervical cord involvement. Linear regression models were used to estimate age- and gender-adjusted group differences between clinical phenotypes after 30 years, and stepwise selection to determine associations between a large sets of MRI predictor variables and physical and cognitive outcome measures. At the 30-year follow-up, the greatest differences in MRI measures between SPMS and RRMS were the number of cortical lesions, which were higher in SPMS (the presence of cortical lesions had 100% sensitivity and 88% specificity), and grey matter volume, which was lower in SPMS. Across all subjects, cortical lesions, grey matter volume and cervical cord volume explained 60% of the variance of the Expanded Disability Status Scale; cortical lesions alone explained 43%. Grey matter volume, cortical lesions and gender explained 43% of the variance of Timed 25-Foot Walk. Reduced cortical magnetization transfer ratios emerged as the only significant explanatory variable for the symbol digit modality test and explained 52% of its variance. Cortical involvement, both in terms of lesions and atrophy, appears to be the main correlate of progressive disease and disability in a cohort of individuals with very long follow-up and homogeneous disease duration, indicating that this should be the target of therapeutic interventions.

## Teaser

Haider et al. followed a cohort of individuals for 30 years after their initial presentation with symptoms suggestive of multiple sclerosis. Cortical involvement was the main correlate of progressive disease and disability and therefore might provide a promising diagnostic and therapeutic target.

## Introduction

Clinical outcomes in multiple sclerosis are highly variable, develop over decades and to date no biomarker has been proven to robustly explain levels of disability, or to reliably distinguish between relapsing remitting (RRMS) and secondary progressive (SPMS) disease. In part this is likely due to previous studies assessing patients who were at different points in their multiple sclerosis disease course or were studied only for a short period of follow-up. Studies comparing patients with disease durations of less than two decades are likely to be hampered by a proportion of patients in the RRMS group who will eventually go on to develop SPMS,^1^ and therefore may already display biomarker features of progressive disease. In cross-sectional studies comparing RRMS and SPMS disease duration is usually not matched, thereby making it difficult to determine whether a given biomarker is associated with disease subtype independently of disease duration.^2^

We recently completed a longitudinal prospective 30-year follow-up of patients recruited soon after a clinically isolated syndrome (CIS) suggestive of multiple sclerosis, to determine the long-term clinical outcomes and their relationship with brain lesions within the first 5 years.^3^ In the 120 patients with known outcome, around a third remained classified as having had a CIS, a third developed SPMS or died because of multiple sclerosis, while the rest had RRMS; of the RRMS group, ∼90% of patients were able to walk without major limitations.^3^ Of those who developed SPMS, nearly all converted within 20 years after symptom onset.

In the present study, we aimed to determine the MRI correlates of progressive disease and disability after 30 years. In this cohort, RRMS and SPMS are well matched for disease duration and given the 30-year follow-up, the RRMS subgroup is likely to be phenotypically relatively ‘pure’, containing few patients who have yet to develop SPMS.

Numerous brain and spinal cord MRI metrics have been proposed to explain disability levels in multiple sclerosis^4^ and measures of cortical^5^ and spinal cord involvement^6^ were most consistently found among the correlates of present and predictors of future disability. Limitations of those previous investigations include the analysis of individual factors, both neurologically and radiologically, as well as with limited duration of follow-up.

We thus set out to use multi-parametric MRI in a CIS cohort studied 30 years after presentation to determine to what extent MRI measures, including white matter lesions, cortical lesions, grey matter volumes, magnetization transfer ratios (MTRs) and cervical spinal cord volumes, distinguish RRMS from SPMS and explain a broad variety of neurological and cognitive outcomes.

## Materials and methods

### Study cohort

This study is based on the analysis of 30-year follow-up clinical and MRI data of a cohort of patients with a CIS, which have been described before.^3^ Briefly, 132 patients with a CIS suggestive of multiple sclerosis were prospectively recruited between 1984 and 1987 at the National Hospital of Neurology and Neurosurgery, and Moorfields Eye Hospital and followed over 30 years. At 30 years, clinical outcome data were obtained in 120 participants,^3^ of whom 63 had MRI data, and they are the subject of this report. The 2010 McDonald MS diagnostic criteria were used.^7^ Twenty-seven patients developed RRMS, 15 SPMS and 21 experienced no further neurological events. As recruitment predated the disease modifying treatment era, the cohort was largely untreated. Only nine (14%) had a disease modifying treatment at any point, all of which were first‐line injectable drugs, with the earliest beginning 10 years after multiple sclerosis diagnosis (when disease modifying treatments first became available in the UK). Of these, five had SPMS at 30 years, and four had RRMS.^3^

### Clinical assessment

Disability was assessed using the Extended Disability Status Scale (EDSS), the timed 25-foot walk (T25FWT) and the 9-Hole Peg Test (9HPT) of the non-dominant hand. Cognitive outcome scores included the paced auditory serial addition test (PASAT), which is a subtest of the Multiple Sclerosis Functional Composite Score,^8^ as well as the Brief International Cognitive Assessment For Multiple Sclerosis (BICAMS) scores^9^^,^^10^ with its three components: The Revised Brief Visuospatial Memory Test (BVMTR), the Symbol Digit Modalities Test (SDMT), and the California Verbal Learning Test (CVLT). BICAMS *z*-scores were obtained in 60 patients with z-scores (adjusted for age, sex and years of education according to the population data from the BICAMS consortium^10^) thus excluding in total five CIS, six RRMS and seven SPMS cases older than 65 years from this subanalysis.

This study was approved by our institutional ethics committee and the National Research Ethics Service (15/LO/0650). Participants gave informed written consent.

### Image acquisition

MRI was performed using a 3 T Achieva system (Philips Healthcare) and a 32-channel receive head coil.

The scan protocol included a 3 D fluid attenuated inversion recovery (FLAIR) acquired in the sagittal plane with repetition/inversion time: 4800/1650 ms and echo time: 297 ms, a voxel size of 1 × 1 × 1 mm^3^. T_2_-weighted axial scans were acquired with repetition time: 4375 ms, echo time: 85 ms, and a voxel size of 0.5 × 0.5 × 3 mm^3^. 3 D T_1_-weighted images were acquired sagitally using a fast field echo (FFE) sequence with repetition/echo time: 7.1/3.2 ms; inversion time: 848 ms, flip angle (α) = 8° and a voxel size of 1 × 1 × 1 mm^3^, covering also the cervical spinal cord. Magnetization transfer ratio (MTR) was calculated based on a Three dimensional slab-selective FFE sequence with two echoes (repetition time: 6.5 ms, echo time 1/echo time 2: 2.8/4.4 ms, α  =  9°), acquired with and without a sinc-Gaussian shaped magnetization transfer prepulse of nominal α  =  360°, offset frequency 1 kHz, duration of 16 ms and a voxel size of 1 × 1 × 1 mm^3^. We used a turbo field echo (TFE) readout (echo train length of four, TFE shot interval 32.5 ms), total time between successive magnetization transfer pulses: 50 ms. Phase-sensitive inversion recovery (PSIR) was acquired axially with a repetition/inversion time of 7302/400 ms and echo time of 13 ms, and a voxel size of 0.5 × 0.5 × 2 mm^3^.

### Image analysis

N4-bias field correction of T_1_-weighted scans was performed to reduce intensity inhomogeneity.^11^ White matter lesion segmentation was performed automatically with Bayesian model selection^12^ using jointly FLAIR and 3 D-T_1_ images and manually edited (L.H.) using the 3 D-Slicer.^13^ Additionally, manual lesion counting (using JIM, version 6, Xinapse Systems) was performed for infratentorial, juxtacortical, deep white matter and periventricular white matter lesions by raters (K.C., F.B.) blinded to the clinical status. T_1_-hypointense white matter lesions were filled with a multi-modality non-local mean algorithm with the most plausible texture.^14^ Thereafter, brain parenchymal fraction, grey matter fraction and thalamic volume (corrected for the total intracranial volume) were computed using an atlas-based segmentation method.^15^

MTR maps were calculated using the following equation:

(1) MTR (in percentage units)=[(MTRoff-MTRon)/MToff)×100]

We report the average cortical MTR derived from the inner and the outer cortical bands that were generated as reported previously.^16^ Similarly, the brain white matter was segmented into 12 concentric bands, based on the distance between the ventricular walls and the cortex and the mean MTR values were calculated in each band.^17^ As reported previously, the first and last bands (nearest to the ventricular and cortical surfaces) were excluded from further analysis to control for partial volume effects.^18^ Within the normal appearing white matter, a gradient was calculated via the equation (MTR in band 3 to MTR in band 1)/2.^17^^,^^18^ Within white matter lesions the average lesional MTR per individual is reported (see Supplementary Fig. 1 for details).

Lesions with clear morphological evidence of cortical involvement^19^ were manually counted on PSIR using 3 D slicer software in consensus blinded to the clinical status (L.H., O.G.). In case of disagreement, decision was reached by expert opinion (F.B.). White matter lesions counts, marked in consensus (K.K.C. with F.B., D.T.C., or both), were available from our earlier analysis.^3^

The cervical spinal cord volume (was measured on T_1_-weighted brain scans with the cord finder tool in JIM (version 6, Xinapse Systems), using an active surface model without straightened cord.^20^ Seed points were manually placed in the centre of the cord starting at the C3/4 disk level and continued consecutively rostral for the next adjacent 40 slices (1 mm slice thickness). The segmentation was reviewed for accuracy and manually edited when necessary (L.H.) and was successful in 57/63 individuals. Cervical spinal cord lesions were not assessed as they are rarely seen on T_1_-weighted images (such as those used to measure cord atrophy in this study) and the T_2_-weighted brain images did not include the cervical cord.

### Statistical analysis

Group differences in the distribution of clinical outcome variables and MRI biomarkers between the 30-year outcome defined groups were estimated with linear regression models adjusting for age and gender. Lesion counts were not corrected for head size. The beta with 95% confidence interval (CI), its corresponding *P*-value, the explained variance (R^2^) and the overall *P*-value are reported.

Linear regression models were computed to explain clinical outcome measures at 30 years based on MRI metrics at 30 years. To allow a comparison of the effect size of different MRI biomarkers [e.g. (*n*) lesion counts versus (ml) volume] on a given clinical outcome measure, standard scores were computed as the fractional number of standard deviations, by which each observed value, e.g. a lesion count, is above or below the mean value of the whole group. Stepwise selection models (i.e. sequential replacement) were used for parameter selection. This process begins without predictors and iteratively adds the most contributing predictors (based on the Akaike information criterion) until the improvement is no longer statistically significant (comparable to forward selection). However, for each new variable added, variables that no longer provide an improvement in the model fit are also eliminated (comparable to backward selection). Cervical spinal cord volume and thalamic volume were corrected for total intracranial volume.

MRI and clinical outcome scores are provided as means with standard deviations (SD) or as medians with 25% to 75% range, as appropriate. Statistical estimates are reported with a 95% CI and the corresponding *P*-value (two-tailed, and exact until *P < *0.001) for this estimate.

The statistical analysis was performed with R-studio.^21^*P*-values < 0.05 (two-tailed) were considered statistically significant.

### Data availability

Anonymized data, not published in the article, will be shared on reasonable request from a qualified investigator.

## Results

### Clinical characteristics

**Figure 1:**
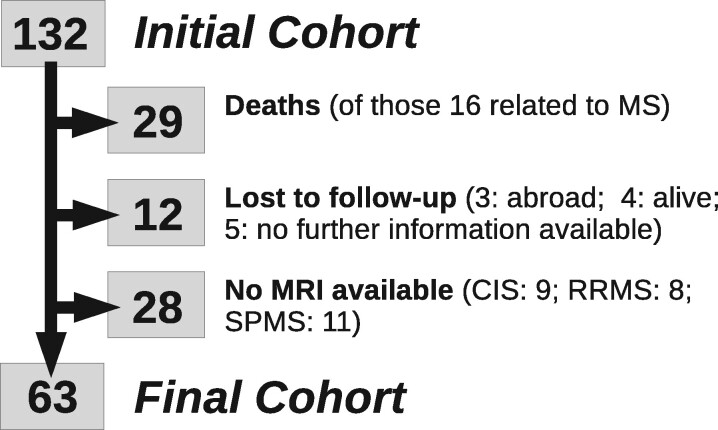
**Flow chart.** Over the course of 30 years, 29 individuals died (of those, 16 was related to multiple sclerosis), 12 were lost to follow-up (three abroad, four alive and five without further information available) and no MRI could be obtained in 28 participants (of whom nine were classified as CIS, eight as RRMS and 11 as SPMS).

Of the initial 132 study participants with a CIS, 29 died during the 30-year follow-up, 16 of them due to multiple sclerosis. From the remaining 103 individuals, 91 were assessed clinically at 30 years. Among those, no MRI could be obtained in 28 subjects (nine CIS, eight RRMS, 11 SPMS), resulting in a final study cohort of 63 (Fig. 1).

**Figure 2:**
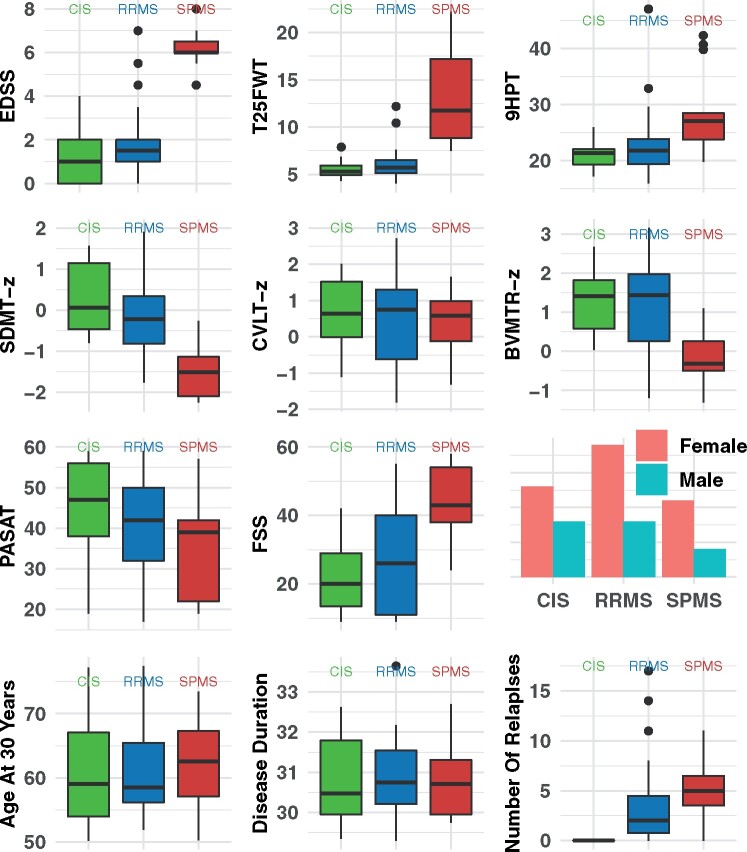
**Clinical metrics by clinical phenotype.** Box plots show the distribution of EDSS, T25FWT, 9HPT, SDMT-z, CVLT-z, BVMTR-z and PASAT measures by 30-year outcome groups. CIS = green; RRMS = blue, SPMS = red. CVLT = California Verbal Learning Test; FSS = Fatigue Severity Scale; PASAT = Paced Auditory Serial Addition Test.

The demographic and clinical characteristics of the 21 CIS, 27 RRMS and 15 SPMS patients are visualized in Fig. 2 and summarized along with their quantitative group differences, obtained from regression models in Table 1 (see also Chung *et al*.^3^ for more details).

**Table 1:** Clinical metrics by clinical phenotype

	CIS (*n = *21)	RRMS (*n = *27)	SPMS (*n = *15)	CIS versus RRMS^a^	CIS versus SPMS^b^	RRMS versus SPMS^c^
Age	60.5 (SD: 7.1)	60.6 (SD: 6.4);	61.9 (SD: 6.7)	Beta: 0.70 (95% CI: −0.36 to 0.17); *P = *0.720	Beta: 2.5 (95% CI: −3.4 to 6.3); *P = *0.559	Beta: 0.87 (95% CI: −3.25 to 4.99); *P = *0.68
Female: Male	13:8	19:8	11:4	Beta: −0.092 (95% CI: −3.11 to 3.89); *P = *0.502	Beta: 0.17 (95% CI: −0.4 to 0.2); *P = *0.462	Beta: −0.02 (95% CI: −0.32 to 0.27); *P = *0.877
Disease duration	30.7 (SD: 1.0)	30.9 (SD: 1.0)	30.8 (SD: 0.9)	Beta: 0.04 (95% CI: −0.55 to 0.64); *P = *0.891	Beta: −0.17 (95% CI: −0.82 to 0.47); *P = *0.598	Beta: −0.15 (95% CI: −0.73 to 0.44); *P = *0.626
*n* relapses years 0–30	0 (SD: 0); NA: 2	3.6 (SD: 4.5); NA: 3	4.7 (SD: 2.9)	Beta: 3.84 (95% CI: 1.67 to 6); *P = *0.001^*^	Beta: 4.76 (95% CI: 3.38 to 6.13); *P < *0.001^*^	Beta: 1.14 (95% CI: −1.53 to 3.82); *P = *0.409
EDSS_30 (#)	1.0 [0.0 to 2.0]	1.5 [1.0 to 2.0]	6.0 [6.0 to 6.5]	Beta: 0.84 (95% CI: −0.02 to 1.69); *P = *0.062	Beta: 5.16 (95% CI: 4.48 to 5.84); *P < *0.001	Beta: 4.41 (95% CI: 3.55 to 5.26); *P < *0.001^*^
T25FWT	5.9 (SD: 1.9)	12 (SD: 32.3)	42.8 (SD: 59.0)	Beta: 7.46 (95% CI: −7.53 to 22.46); *P = *0.335	Beta: 45.34 (95% CI: 24.07 to 66.62); *P < *0.001^*^	Beta: 33.21 (95% CI: 7.82 to 58.6); *P = *0.014^*^
9HPT	21.4 (SD: 3.4)	23.9 (SD: 4.8)	36.9 (SD: 15.0)	Beta: 3.11 (95% CI: 0.91 to 5.3); *P = *0.008^*^	Beta: 17.18 (95% CI: 11.18 to 23.18); *P < *0.001^*^	Beta: 13.41 (95% CI: 8.36 to 18.45); *P < *0.001^*^
Fatigue	21.5 (SD: 10); NA: 1	27.6 (SD: 14.9); NA: 2	44.1 (SD: 10.3); NA: 2	Beta: 6.23 (95% CI: −1.71 to 14.16); *P = *0.132	Beta: 21.68 (95% CI: 14.23 to 29.13); *P < *0.001^*^	Beta: 16.83 (95% CI: 7.59 to 26.08); *P = *0.001^*^
BVMTR-z	1.3 (SD: 0.8); NA: 5	1.2 (SD: 1.1); NA: 6	−0.1 (SD: 0.8); NA: 7	Beta: −0.13 (95% CI: −0.75 to 0.49); *P = *0.691	Beta: −1.45 (95% CI: −2.2 to −0.7); *P = *0.001^*^	Beta: −1.41 (95% CI: −2.31 to −0.51); *P = *0.005^*^
CVLT-z	0.7 (SD: 0.9); NA: 5	0.5 (SD: 1.2); NA: 5	0.4 (SD: 1.0); NA: 7	Beta: −0.16 (95% CI: −0.72 to 0.4); *P = *0.583	Beta: −0.32 (95% CI: −1.11 to 0.47); *P = *0.44	Beta: −0.2 (95% CI: −0.89 to 0.5); *P = *0.583
SDMT-z	0.3 (SD: 0.9); NA: 5	−0.2 (SD: 0.9); NA: 6	−1.5 (SD: 0.7); NA: 7	Beta: −0.58 (95% CI: −1.16 to 0); *P = *0.057	Beta: −1.79 (95% CI: −2.47 to −1.1); *P < *0.001^*^	Beta: −1.24 (95% CI: −1.98 to −0.5); *P = *0.003^*^
PASAT	46.5 (SD: 11.5); NA: 2	41.2 (SD: 10.9); NA: 2	35.7 (SD: 13.7); NA: 7	Beta: −5.49 (95% CI: −12.38 to 1.4); *P = *0.126	Beta: −9.82 (95% CI: −18.39 to −1.24); *P = *0.033^*^	Beta: −5.4 (95% CI: −13.65 to 2.86); *P = *0.209

Clinical metrics are summarized as mean (SD) within the 30-year clinical outcome defined groups: CIS, RRMS and SPMS. EDSS(#) values at 30 years are provided with median and [25%–75% range]. Comparisons between CIS, RRMS and SPMS were performed using linear regression models adjusting for age and sex. We report the Beta coefficients with their 95% CI and *P*-value. Exact *P*-values are provided until *P < *0.001. If data-points were not available/missing, the number is provided). CVLT = California Verbal Learning Test; dom./nondom. hand = dominant/non-dominant hand; NA = not available/missing; PASAT = Paced Auditory Serial Addition Test.

a CIS = 0, RRMS = 1.

b CIS = 0, SPMS = 1.

c RRMS = 0, SPMS = 1.

* *P*-value significance (*P *<* *0.05).

Adjusting for age and sex, the distributions of clinical outcome measures were well separated between RRMS and SPMS cases, whilst they were largely overlapping between CIS and RRMS, with the exception of the 9HPT, which was more abnormal in RRMS than CIS (Fig. 2 and Table 1). SPMS showed worse scores for the EDSS, T25FWT, and 9HPT (*P < *0.001) than RRMS (Table 1). Differences between RRMS and SPMS were also found for cognitive outcome measures, including the revised BVMTR-*z* and SDMT-*z*, which showed a greater abnormality in SPMS than RRMS (Table 1). No differences in the cognitive tests were seen between RRMS and CIS.

### MRI characteristics

**Figure 3:**
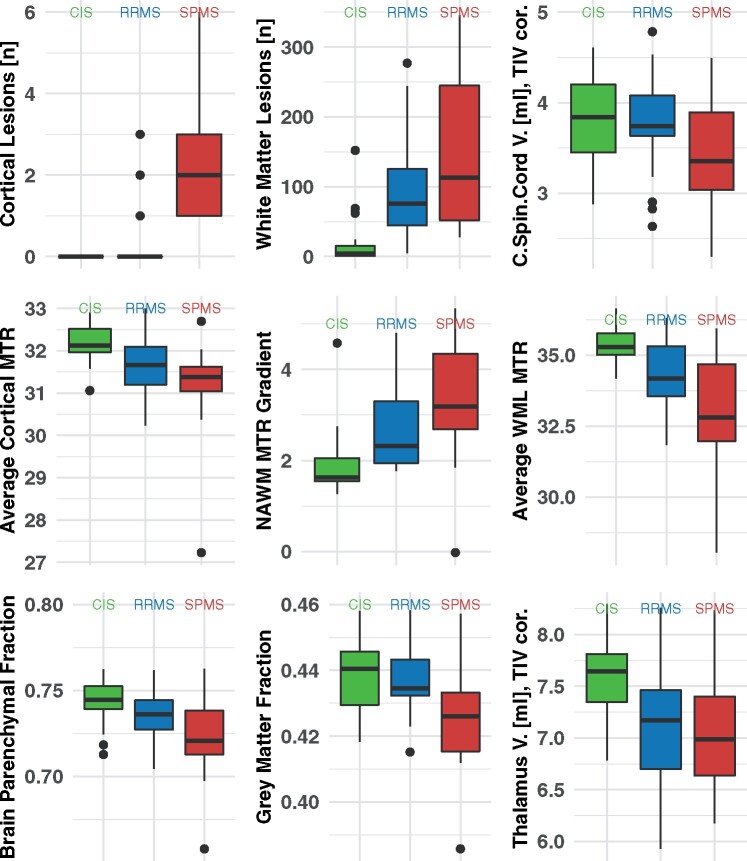
**MRI metrics by clinical phenotypes.** Box plots show the distribution of cortical lesions, white matter lesions, cervical spinal cord volume, average cortical MTR, average MTR in white matter lesions, the MTR gradient towards the ventricular surface within the normal appearing white matter, brain parenchymal and grey matter fraction as well as the thalamic volume. CIS = green; RRMS = blue; SPMS = red. C.Spin.Cord V. = cervical spinal cord volume; NAWM = normal-appearing white matter; TIV cor. = total intracranial volume corrected; WML = white matter lesion.

The distribution of quantitative MRI measures within the 30-year outcome defined groups (CIS, RRMS and SPMS) are visualized in Fig. 3 and summarized with their quantitative group differences, obtained from regression models, in Table 2 (a version based on *z*-scores is available in Supplementary Table 3).

**Table 2:** MRI metrics by clinical phenotype

	CIS (*n = *21)	RRMS (*n = *27)	SPMS (*n = *15)	CIS versus RRMS (R^2^; *P*)^a^	CIS versus SPMS (R^2^; *P*)^b^	RRMS versus SPMS (R^2^; *P*)^c^
Cortical lesions, *n*	0 (SD: 0); NA: 2	0.2 (SD: 0.7); NA: 3	2.2 (SD: 1.5)	R^2^ = 0.06; *P = *0.479	R^2^ = 0.56; *P < *0.001^*^	R^2^ = 0.46; *P < *0.001^*^
				Beta: 0.24 (95% CI: −0.08 to 0.56); *P = *0.15	Beta: 2.22 (95% CI: 1.53 to 2.91); *P < *0.001^*^	Beta: 1.99 (95% CI: 1.29 to 2.69); *P < *0.001^*^
White matter lesions, *n*	12 (SD: 19.7); NA: 1	94 (SD: 69.1); NA: 2	155 (SD: 106.3)	R^2^ = 0.32; *P = *0.001^*^	R^2^ = 0.47; *P < *0.001^*^	R^2^ = 0.14; *P = *0.133
				Beta: 75.88 (95% CI: 42.58 to 109.17); *P < *0.001^*^	Beta: 138.29 (95% CI: 87.52 to 189.06); *P < *0.001^*^	Beta: 62.5 (95% CI: 8.64 to 116.36); *P = *0.029^*^
Cervical spinal cord CSA, mm^2^	3.836 (SD: 0.561); NA: 5	3.794 (SD: 0.534); NA: 4	3.457 (SD: 0.612)	R^2^ = 0.15; *P = *0.11	R^2^ = 0.42; *P = *0.002	R^2^ = 0.35; *P = *0.001^*^
				Beta: −0.072 (95% CI: −0.389 to –0.244); *P = *0.657	Beta: −0.415 (95% CI: −0.751 to −0.079); *P = *0.022^*^	Beta: −0.335 (95% CI: −0.649 to −0.021); *P = *0.043^*^
MTR cortex	32.16 (SD: 0.5); NA: 5	31.68 (SD: 0.7); NA: 4	31.13 (SD: 1.2)	R^2^ = 0.34; *P = *0.001^*^	R^2^ = 0.29; *P = *0.026^*^	R^2^ = 0.23; *P = *0.022^*^
				Beta: −0.52 (95% CI: −0.869 to −0.167); *P = *0.006^*^	Beta: −1.058 (95% CI: −1.721 to −0.396); *P = *0.004^*^	Beta: −0.53 (95% CI: −1.083 to 0.023); *P = *0.069
MTR WML	35.39 (SD: 0.7); NA: 3	34.3 (SD: 1.2); NA: 3	32.97 (SD: 2.3)	R^2^ = 0.4; *P < *0.001	R^2^ = 0.37; *P = *0.006	R^2^ = 0.22; *P = *0.024^*^
				Beta: −1.199 (95% CI: −1.795 to −0.602); *P < *0.001^*^	Beta: −2.471 (95% CI: −3.707 to −1.235); *P = *0.001^*^	Beta: −1.329 (95% CI: −2.366 to −0.292); *P = *0.017^*^
NAWM MTR gradient	1.92 (SD: 0.8); NA: 3	2.69 (SD: 0.9); NA: 3	3.24 (SD: 1.3)	R^2^ = 0.19; *P = *0.034^*^	R^2^ = 0.33; *P = *0.009	R^2^ = 0.07; *P = *0.413
				Beta: 0.808 (95% CI: 0.284 to 1.331); *P = *0.004^*^	Beta: 1.228 (95% CI: 0.483 to 1.972); *P = *0.003^*^	Beta: 0.544 (95% CI: −0.15 to 1.238); *P = *0.133
Brain parenchymal fraction	0.75 (SD: 0.012); NA: 1	0.74 (SD: 0.012); NA: 2	0.72 (SD: 0.025)	R^2^ = 0.39; *P < *0.001^*^	R^2^ = 0.36; *P = *0.002^*^	R^2^ = 0.2; *P = *0.037^*^
				Beta: −0.009 (95% CI: −0.015 to −0.003); *P = *0.004^*^	Beta: −0.022 (95% CI: −0.034 to −0.01); *P = *0.001^*^	Beta: −0.013 (95% CI: −0.024 to −0.002); *P = *0.021^*^
Grey matter fraction	0.44 (SD: 0.011); NA: 1	0.44 (SD: 0.01); NA: 2	0.42 (SD: 0.016)	R^2^ = 0.54; *P < *0.001^*^	R^2^ = 0.5; *P < *0.001^*^	R^2^ = 0.37; *P < *0.001^*^
				Beta: −0.002 (95% CI: −0.006 to 0.002); *P = *0.334	Beta: −0.014 (95% CI: −0.021 to −0.006); *P = *0.001^*^	Beta: −0.011 (95% CI: −0.018 to −0.004); *P = *0.003^*^
Thalamus V., ml	7.591 (SD: 0.408); NA: 1	7.09 (SD: 0.619); NA: 2	7.086 (SD: 0.602)	R^2^ = 0.23; *P = *0.008^*^	R^2^ = 0.25; *P = *0.027^*^	R^2^ = 0.16; *P = *0.074
				Beta: −0.537 (95% CI: −0.844 to −0.230); *P = *0.001^*^	Beta: −0.542 (95% CI: −0.879 to −0.204); *P = *0.004^*^	Beta: −0.04 (95% CI: −0.404 to –0.323); *P = *0.828

Radiological variables are summarized as mean (SD) within the 30-year clinical outcome defined groups: CIS, RRMS and SPMS. Comparisons between CIS, RRMS and SPMS were performed using linear regression models adjusting for age and sex. We report the beta coefficients with their 95% CI and *P*-value, as well as the overall model fit given as the R^2^ and overall *P*-value. Exact *P*-values are provided until *P < *0.001.

NA = not available (i.e. number of missing observations); NAWM = normal appearing white matter; WML = white matter lesions.

a CIS = 0, RRMS = 1.

b CIS = 0, SPMS = 1.

c RRMS = 0, SPMS = 1.

* *P*-value significance (*P *<* *0.05).

Adjusting for age and sex, the greatest difference between RRMS and SPMS was present in the number of cortical lesions, followed by grey matter fraction. Cortical lesions were seen in 3 of 27 RRMS patients (mean 0.2, SD 0.7) and in all SPMS (*n *=* *15, mean 2.2, SD 1.5) patients. Of note, the three RRMS patients with cortical lesions had the highest EDSS scores among all RRMS [median EDSS 1.5 (25% to 75%, range 1.0–2.0) (Fig. 3, Table 2 and Supplementary Table 3).

Adjusting for age and sex, the greatest difference between CIS and RRMS were observed in higher white matter lesion counts in RRMS, followed by lower lesional MTR in RRMS. The mean white matter lesion count in individuals with CIS was 12 (SD 19.7), compared to 94 (SD 69.1) in RRMS, (*P < *0.001) (Fig. 3, Table 2 and Supplementary Table 3).

**Figure 4:**
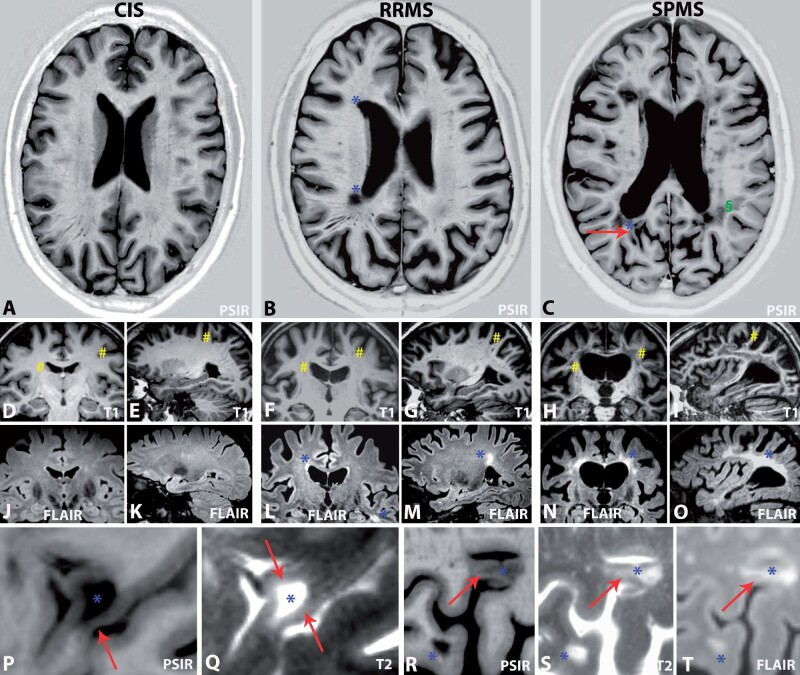
**Typical MRI patterns of CIS, RRMS and SPMS.** (**A**, **D**, **E**, **J** and **K**) A 52-year-old female with CIS (EDSS = 0.0). (**A**, **J** and **K**) No white matter or cortical lesions; (**A**, **D** and **E**) slender ventricles, and no sulcal widening (hash symbol in **D** and **E**), brain parenchymal fraction = 0.755. (**B**, **F**, **G**, **L** and **M**) A 67-year-old male with RRMS (EDSS = 1.0). Note the multiple sclerosis typical periventricular lesions, suggestive of a perivenous distribution (asterisk in **B** and **M**) and typical involvement of the temporal lobes (asterisk in *lower right corner* of **L**) as well as unspecific periventricular caps (*upper left* asterisk in **L**), but no cortical lesions. Moderate ventricular and sulcal widening was observed on T_1_-weighted images (hash symbol in **F** and **G**), brain parenchymal fraction: 0.727. (**C**) A 66-year-old female with SPMS (EDSS = 6.5) with leukocortical (arrow in **C**) and juxtacortical white matter lesions (asterisk and arrow in **C**) and numerous non-specific deep white matter lesions (section symbol in **C**). Moderate ventricular and sulcal widening (hash symbol in **H** and **I**), brain parenchymal fraction: 0.727. (**H**, **I**, **N** and **O**) A 53-year-old male with SPMS (EDSS = 6.5) revealed diffuse and widespread signal alterations that affected large portions of the white matter (asterisk in **N** and **O**). Severe widening of the inner and outer CSF spaces (hash symbol in **H** and **I**), brain parenchymal fraction: 0.658. (**P** and **Q**) A 75-year-old female with SPMS (EDSS = 5.5). High magnification images show a leukocortical multiple sclerosis lesion (asterisk in **P** and **Q**) with extension of white matter signal alterations into the adjacent cortex (arrow in **P** and **Q**) and associated cortical atrophy (arrow in **P**). (**R**–**T**) A 57-year-old female with RRMS (EDSS = 2.5). Leukocortical lesions (*top* asterisk and arrows in **R**–**T**) are distinct from juxtacortical lesions (*bottom* asterisk in **R**–**T**), which (based on the available sequences) abut the cortical surface and show no morphological evidence for cortical involvement. Supplementary Table 2 contains all clinical and quantitative MRI biomarker details.

Graphical examples of the typical MRI patterns that emerged are shown in Fig. 4.

### Explaining physical and cognitive impairment by MRI

Adjusting for age and sex, linear regression models with stepwise selection were performed on *z*-scores, to allow the effect sizes of different variables to be compared amongst each other.

Cortical lesion counts (beta: 0.37, 95% CI: 0.23 to 0.508), cervical spinal cord volume (beta: −0.27, 95% CI: −0.421 to −0.109), grey matter volume (beta: −0.26, 95% CI: −0.444 to −0.074) collectively explained 60% (R^2^) of the variance of the EDSS (Table 3). Forcing the model to include only cortical lesions, 43% (R^2^) of the EDSS could be explained. Models with only one factor are reported in Supplementary Table 2. Similarly, cortical lesion counts (beta: 0.03, 95% CI: 0.004 to 0.055), grey matter volume (beta: −0.06, 95% CI: −0.096 to −0.033) and sex (beta: −0.04, 95% CI: −0.079 to −0.01; female = 0, male = 1) collectively explained 43% (R^2^) of the variance of T25FWT. On the contrary, grey matter MRI measures did not explain 9HPT, whilst MTR of white matter lesions (beta: −0.26, 95% CI: −0.363 to −0.163) and the cervical cord volume (beta: −0.17, 95% CI: −0.288 to −0.06) explained 49% (R^2^) of the variance in 9HPT.

**Table 3:** Linear regression models for explanation of clinical outcomes via MRI biomarker, adjusting for age and gender

	Selected variables	MRI biomarker				
**EDSS**		**Cortical lesions**	**Cervical cord volume**	**GMF**	**BPF**	
	Estimate (95% CI)	0.37 (0.23 to 0.508)**	−0.27 (−0.421 to −0.109)*	−0.26 (−0.444 to −0.074)*	0.17 (−0.041 to 0.383)	−
	Overall	R^2^: 0.60; AIC.:72; *P *<* *0.001				
**T25FWT**		**GMF**	**Cortical lesions**	**Sex (female = 0, male = 1)**	**–**	**–**
	Estimate (95% CI)	−0.06 (−0.096 to −0.033)**	0.03 (0.004 to 0.055)*	−0.04 (−0.079 to −0.01)*	–	–
	Overall	R^2^: 0.43; AIC.: 83; *P *<* *0.001				
**9HPT**		**MTR WML (avg)**	**Cervical cord volume**	**–**	**−**	**–**
	Estimate (95% CI)	−0.26 (−0.363 to −0.163)**	−0.17 (−0.288 to −0.06)*	–	–	–
	Overall	R^2^: 0.49; AIC.:41; *P *<* *0.001				
**SDMT-z**		**MTR Cortex (avg)**	**Cortical lesions**	**White matter lesions**	**BPF**	**Cervical spinal cord volume**
	Estimate (95% CI)	0.87 (0.257–1.482)*	−0.27 (−0.643 to 0.105)	−0.31 (−0.642 to 0.023)	−0.5 (−1.059 to 0.06)	0.26 (−0.048 to 0.567)
	Overall	R^2^: 0.52; AIC : 89; *P *<* *0.001				
**CVLT-z**		**MTR Cortex (avg)**	**MTR WML (avg)**	**GMF**	**Cervical spinal cord volume**	**–**
	Estimate (95% CI)	0.63 (0.066 to 1.203)*	−0.31 (−0.728 to 0.117)	0.29 (−0.116 to 0.687)	0.25 (−0.04 to 0.538)	–
	Overall	R^2^: 0.34; AIC.:98; *P *=* *0.010				
**BVMTR-z**		**MTR Cortex (avg)**	**White matter lesions**	**Cervical cord volume**	**BPF**	**Thalamus volume**
	Estimate (95% CI)	0.77 (0.143 to 1.393)*	−0.5 (−0.811 to −0.195)*	0.42 (0.104 to 0.736)*	−0.42 (−0.997 to 0.16)	−0.26 (−0.588 to 0.059)
	Overall	R^2^: 0.49; AIC: 92; *P *<* *0.001				
**PASAT**		**MTR Cortex (avg)**	**MTR NAWM (gradient)**	**BPF**	**–**	**–**
	Estimate (95% CI)	0.77 (0.256 to 1.292)*	−0.3 (−0.673 to 0.07)	−0.47 (−1.053 to 0.114)	–	–
	Overall	R^2^: 0.26; AIC: 126; *P *=* *0.006				

Stepwise selection was used to determine linear regression models that explain the different clinical outcomes via MRI biomarker including age and gender as covariates. AIC = Akaike information criterion; BPF = brain parenchymal fraction; CVLT = California Verbal Learning Test; GMF = grey matter fraction; PASAT = paced auditory serial addition test.

* *P *<* *0.05.

** *P *<* *0.001.

When considering the cognitive outcomes, cortical MTR emerged as the strongest significant predictive factor in all models to explain cognitive outcome measures (SDMT, CVLT, BVMTR, PASAT). In particular, cortical MTR (beta: 0.87, 95% CI: 0.257 to 1.482) was the only explanatory variable that remained statistically significant and explained up to 52% (R^2^) of the variability of SDMT, with estimates of cortical lesions and estimates above the significance level for white matter lesions, brain parenchymal fraction and cervical spinal cord volume above the statistical significance level (Table 3). A model of the SDMT forced to include only cortical MTR explained 16% (R^2^) (Supplementary Table 2). Similarly, cortical MTR explained 34% (R^2^) of the variance of the CVLT (beta: 0.63, 95% CI: 0.066 to 1.203), with estimates above the significance level for MTR in white matter lesions, grey matter volume and cervical cord volume. Similarly, the model for the revised BVMTR, which was explained for 49% (R^2^), included cortical MTR (beta: 0.77, 95% CI: 0.143 to 1.393), white matter lesion counts (beta: −0.5, 95% CI: −0.811 to −0.195) and cervical spinal cord volume (beta: 0.42, 95% CI: 0.104 to 0.736). Finally, the PASAT was explained for 26% (R^2^) by cortical MTR (beta: 0.77, 95% CI: 0.256 to 1.292).

## Discussion

In the present cohort, despite 30 years of follow-up, 43% remained phenotypically classified as having RRMS with disability levels not too different from those who remained CIS (Table 1), raising the question of which factors discriminate SPMS from RRMS. In this unique cohort with a long and homogenous disease duration, we found that cortical lesions were the clearest determinant of a progressive disease course, and that cortical involvement 30 years after symptom onset was the dominant factor explaining both physical and cognitive outcomes.

Cortical lesions were absent in all individuals who remained CIS, found in three of 27 patients who developed RRMS patients but were present in all 15 individuals affected by SPMS. While greater numbers of cortical lesions have been associated with progression in both MRI^22^ and histopathological studies,^23^ the differences were less distinct compared with the present study. Previous histopathological studies have shown grey matter lesions to be present in acute early^24^ and RRMS^25^ patients. We hypothesize that this can be explained on the one hand by having studied RRMS groups cross-sectionally with short follow-up and disease duration, thus including individuals who would potentially go on to develop SPMS.^26^ On the other hand, subpial demyelination, accounting quantitatively for most of the cortical involvement, as depicted by immunohistochemistry for myelin antigens,^27^ still remains undetected by current clinically available *in vivo* MRI, irrespective of the applied imaging techniques.^28^ Leukocortical lesions, therefore, only reveal the tip of the iceberg. Our lack of sensitivity for detection of cortical lesions in cases with lower cortical lesion loads or primarily subpial demyelination may thus also explain the absence of cortical lesions in RRMS in our present study. Finally, primary cortical demyelination is highly specific for multiple sclerosis,^29^ whereas MRI does detect white matter lesions that are less specific for multiple sclerosis, including leukoariosis (and other) non-multiple sclerosis lesions.^4^^,^^30^ This might be especially relevant in a cohort with an older age, such as the present one.

The assessment of cortical lesions is challenging. Conventional MRI sequences are suboptimal,^19^ thus results differ dependent on which sequence is used.^31^ This comes with a high inter- and intra-rater variability of cortical lesion assessment^4^ and a reliable automatic detection method has not yet been established. We sought to minimize these issues by performing blinded consensus ratings with two experienced readers (L.H. and O.G.), with blinded expert opinion in case of different ratings (F.B.) and restriction of the analysis to obvious lesions as shown by Fig. 4.

In line with previous literature,^32^ we observed a dominant effect of grey matter MTR on cognitive outcome measures, whereas grey matter atrophy was more predictive for EDSS (and T25FWT in our study). Overall, disability measures were frequently explained by grey matter related MRI measures, but additional metrics increased model performances. For example, cervical cord volume was also associated with EDSS, 9HPT, BVMTR; white matter lesions with the BVMTR; and white matter lesion MTR with the 9HPT. We observed weak gender effects for the T25FWT (Table 3). This is in line with previous work, that has suggested a dominant role for cortical involvement for prediction of current^5^ and future disability,^26^ but also reflects the way in which multiple sclerosis pathology at any point within a neural network can cause disability.^33^

It is noteworthy that despite a highly controlled study environment and well separated cohorts with long and homogeneous disease duration, a considerable amount of variance remains unexplained by current structural imaging. Some of this uncertainty might reflect limitations inherent to clinical measurement [e.g. EDSS scores, both inter-operator and intra-operator variability contribute significantly to estimated scores^34^]. However, it also suggests that current structural MRI sequences are relatively insensitive^35^ to biologically and clinically relevant aspects of tissue injury,^36^ and the networked nature of the brain.^37^^,^^38^ For any given neurological or cognitive outcome, some parts of the brain, within a given individual, will play a greater part than others.^39^

Thalamic atrophy has been repeatedly suggested as an early correlate of present and subsequent disability in multiple sclerosis.^40^ While the group differences for thalamic volume in our study were significant comparing CIS and RRMS (*P *=* *0.001) and CIS and SPMS (*P *=* *0.004), RRMS compared with SPMS was far from the significance level (*P *=* *0.828). To some extend the sample size, as discussed below, might have limited our sensitivity to detect group differences. However, given the highly significant differences compared to CIS, our findings might offer additional insights: First, that most studies comparing RRMS and SPMS have not been able to match individuals, i.e. the SPMS groups are often older with longer disease durations, and thus observed differences might be partially a function of time that separates RRMS and SPMS. Second, that thalamic atrophy reaches a floor effect already in RRMS, and that further decline in thalamic volume as a function of age (and disease duration) occurs in both SPMS and RRMS.

This study has several other limitations. We did not have a control group and it has been reported that CIS might harbour residual inflammatory damage when compared with healthy controls,^41^ but this does not affect our study design. At 30 years, 28 participants (nine CIS, eight RRMS and 11 SPMS) for whom we had clinical data did not have an MRI, thus reducing our sample size and statistical power, which is relatively low when compared to other CIS cohorts.^42–44^ A significant caveat is that 16 participants died due to multiple sclerosis during the follow-up, and so even among those with SPMS imaging findings will be biased towards those with a relatively benign disease evolution. Additionally, in 28 subjects with a known 30-year follow-up outcome, no MRI could be obtained (Table 1). Of those, nine were classified as CIS, eight as RRMS and 11 as SPMS. The median EDSS in CIS: 2.0 (25%–75% range: 0–2.0) and RRMS 2 (range: 0.5–2.0) was comparable to subjects who were included in the MRI analysis: CIS: 1 (25%–75% range: 0–2.0), RRMS: 1.5 (25%–75% range: 1.0–2.0). However, the EDSS scores in SPMS subjects for whom no MRI could be obtained were higher than in those with MRI [8.0 (25%–75% range: 6.5–8.5) compared with 6.0 (6.0–6.5)]. While this may have reduced our statistical power to detect group differences due to exclusion of subjects with more severe disease, it is not likely to introduce a systematic bias towards spurious differences being found. Given the average age of participants in this cohort, age-related changes (white matter lesions in particular) and cortical atrophy cannot be robustly separated from multiple sclerosis pathology. This has the potential to complicate our assessments of clinical associations, as age-related white matter lesions may also affect clinical outcomes^45^ or dilute the apparent effect of multiple sclerosis white matter lesions,^33^ which is less likely to be the case for grey matter lesions. Natural ageing is thus likely to be less relevant to associations of cortical pathology with clinical outcomes, as age was homogeneously distributed between the three outcome groups [CIS: 60.5 (SD: 7.1), RRMS: 60.6 (SD: 6.4) and SPMS: 61.9 (SD: 6.7)] and included as a covariate in the statistical models. In the present study we report factors that most robustly distinguish RRMS and SPMS 30 years after CIS. However, due to the study design, we do not know to which extent, or at which time point in the evolution of the disease, such factors become relevant, or if cervical spinal cord volume,^46^ or MRI lesion loads,^47^ would outperform cortical involvement for prediction of conversion or disability progression rates. While we did analyse a large spectrum of structural MRI and clinical outcome measures, we could not evaluate the presence of cervical spinal cord lesions and susceptibility weighted features. Additionally, it is currently a matter of debate how to adjust cervical spinal cord volumes between individual subjects. In the present study, we corrected the cervical spinal cord volumes for the total intracranial volume, assuming, in line with previous research,^6^ a positive association between head size and cord volume. However, there is reason to argue for different adjustment methods, such as patient size and weight, for which we could not control. In the absence of a generally accepted correction method for spinal cord volumes, where due to the small measures even minor adjustments could potentially influence statistical outcomes, our results regarding this metric might be considered preliminary.

In conclusion, in the present cohort we found that cortical involvement was main MRI feature that distinguished SPMS from RRMS. Cortical lesions, grey matter fraction and cortical MTRs, most consistently explained neurological and cognitive impairments, indicating that this should be the target of therapeutic interventions.

## Supplementary Material

awab033_Supplementary_Data

## Abbreviations

9HPT: 9-Hole Peg Test
BVMTR: Brief Visuospatial Memory Test Revised
CIS: clinically isolated syndrome
EDSS: Extended Disability Status Scale
MTR: magnetization transfer ratio
RRMS: relapsing-remitting multiple sclerosis
SDMT: Symbol Digit Modalities Test
SPMS: secondary progressive multiple sclerosis
T25FWT: Timed 25-Foot Walking Test
