# Supplementary material for: Cortical involvement determines impairment 30 years after a clinically isolated syndrome
Source: Brain. 2021 Apr 21;144(5):1384–95. doi: 10.1093/brain/awab033 (PMC8219364; doi:10.1093/brain/awab033)
Supplement: awab033_Supplementary_Data [file awab033_supplementary_data.zip › awab033_suppl-data/brain-2020-01742-File009.pdf]

Supplementary

<u>Supplementary Figure 1</u>

Title: The distribution of MTR values relative to the surface of the brain

Legend:

A) Cortical MTR values were measured in the inner and outer cortical layer (adjacent to the pial surface) as reported previously (Pardini et al., 2016). The differences in inner and outer cortical MTR did not reach the statistical significance with unadjusted non-parametric tests; Inner cortical MTR: RRMS: 34 (33-34) vs. SPMS: 34 (33-34), $p< 0.0939$; Outer cortical MTR: RRMS: 31 (30-31) vs. SPMS: 31 (30-31), $p< 0.3052$). Similarly, there was no difference in the ratio/the gradient between inner and outer cortical MTR (RRMS: 1.4 (1.4-1.5), SPMS: 1.4 (1.4-1.5), $p<0.5386$). We used average cortical MTR values obtained from inner and outer cortical MTR in the main manuscript.

B) MTR values of the normal appearing white matter dropped towards the ventricular surface. The steepness of this decrease (MTR in band 3—MTR in band 1)/2 (Pardini et al., 2016, Brown et al., 2017)) was higher for SPMS: 3.1 (2.6-4.3) than for RRMS: 2.3 (1.9-3.3), $p<0.0458$.

C) While the distribution of MTR values from white matter lesion closer to the ventricle indicates a trend toward lower MTR values within white matter lesions closer to the ventricle, we did not observe an increase in the steepness of this ratio between CIS, RRMS and SPMS (data not shown).

Abbreviations: CIS, RRMS, SPMS

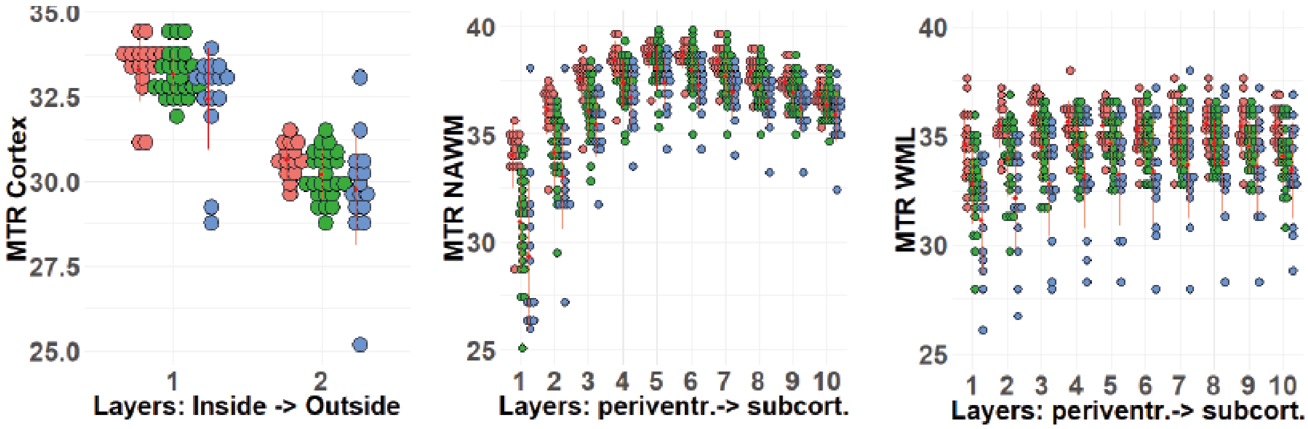

A **B** **C**

<u>Supplementary Table 1</u>

Title: **Clinical and MRI metrics for individuals presented in figure 3**

Legend: All clinical and radiological variables for the six cases presented in figure 3.

Abbreviations: **-**

Supplementary Table 2

Title: Prediction of clinical outcome measures at 30-years based on individual factors only

Legend: This Table shows linear models that were forced, to include only single MRI variables derived from forward selection for prediction of clinical outcome measures.

Abbreviations: CIS: clinically isolated syndrome, RRMS: relapsing remitting multiple sclerosis; SPMS: secondary progressive multiple sclerosis, EDSS: expanded disability status scale; T25FWT: timed 25-foot walk test; 9HPT: 9-hole peg test; SDMT: symbol digit modalities test; CVLT: California verbal learning test; BVMTR: revised brief visuospatial memory test; PASAT: paced auditory serial addition test; GMF: grey matter fraction; BPF: brain parenchymal fraction; cer. spin. v.: cervical spinal cord volume; MTR: magnetization transfer ratio

**Prediction of clinical outcome at 30-years, as measured by:**					
	**EDSS**		**Cortical Lesions**	**cerv. spin. cord V.**	**GMF**
		Estimate (95%CI)	0.45 (0.318 to 0.584)	-0.35 (-0.511 to -0.195)	-0.32 (-0.471 to -0.166)
		Estimate p-value<	p< 0.0001	p< 0.0001	p< 0.0001
		Overall	R2: 0.43; p< 0.0001	R2: 0.26; p< 0.0001	R2: 0.22; p< 0.0001
	**T25FWT**		**GMF**	**Cortical Lesions**	**Gender (f=0, m=1)**
		Estimate (95%CI)	-0.06 (-0.085 to -0.032)	0.05 (0.024 to 0.074)	-0.02 (-0.052 to 0.003)
		Estimate p-value<	p< 0.0001	p< 0.0004	p< 0.092
		Overall	R2: 0.25; p< 0.0001	R2: 0.22; p< 0.0004	R2: 0.05; p< 0.092
	**9HPT**		**MTR WML (avg)**	**cerv. spin. cord V.**	
		Estimate (95%CI)	-0.36 (-0.52 to -0.207)	-0.23 (-0.388 to -0.076)	
		Estimate p-value<	p< 0.0001	p< 0.0052	
		Overall	R2: 0.29; p< 0.0001	R2: 0.15; p< 0.0052	
	**SDMT-z**		**MTR Cortex (avg)**		
		Estimate (95%CI)	0.53 (0.146 to 0.913)		
		Estimate p-value<	p< 0.0101		
		Overall	R2: 0.16; p< 0.0101		
	**CVLT-z**		**MTR Cortex (avg)**		
		Estimate (95%CI)	0.43 (0.036 to 0.819)		
		Estimate p-value<	p< 0.0386		
		Overall	R2: 0.1; p< 0.0386		
	**BVMTR-z**		**MTR Cortex (avg)**	**White M. Lesions**	**cerv. spin. cord V.**
		Estimate (95%CI)	0.68 (0.306 to 1.046)	-0.51 (-0.766 to -0.245)	0.34 (0.033 to 0.645)
		Estimate p-value<	p< 0.0009	p< 0.0004	p< 0.0364
		Overall	R2: 0.25; p< 0.0009	R2: 0.26; p< 0.0004	R2: 0.11; p< 0.0364
	**PASAT**		**MTR Cortex (avg)**		
		Estimate (95%CI)	0.47 (0.218 to 0.719)		
		Estimate p-value<	p< 0.0006		
		Overall	R2: 0.21; p< 0.0006		

Supplementary Table 3

Title: **z-scaled MRI metrics by clinical phenotypes**

Legend: Similar to table to from the main manuscript all three possible group comparisons between CIS, RRMS and SPMS are performed using linear regression models adjusting for age and gender. The data were however z-scaled and the extend of group difference can thus be compared across different units. Beta coefficients with their 95%CI and p-value are reported. Exact p-values are provided until p<0.00001. The coding for groups was 0/1 with: CIS=0/ RRMS=1; CIS=0/ SPMS=1; RRMS=0/ SPMS=1

Abbreviations: CIS: clinically isolated syndrome, RRMS: relapsing remitting multiple sclerosis; SPMS: secondary progressive multiple sclerosis

**CIS vs. RRMS**	**Estimate (Beta)**	**Beta 95% CI**	**P-value**
Cortical Lesions [n]	0.441	0.44 - 0.44	0.1500
White Matter Lesions [n]	1.114	0.63 - 1.6	**0.0000**
Cerv. Spinal Cord V. [ml]	-0.136	-0.73 - 0.46	0.6570
MTR Cortex	-0.789	-1.32 - -0.25	**0.0060**
MTR WML	-1.015	-1.52 - -0.51	**0.0000**
NAWM MTR Gradient	0.879	0.31 - 1.45	**0.0040**
Brain Parenchymal Fraction	-0.727	-1.19 - -0.26	**0.0040**
Grey Matter Fraction	-0.199	-0.6 - 0.2	0.3340
Thalamus V. [ml]	-0.908	-1.43 - -0.39	**0.0000**
**CIS vs. SPMS**			
Cortical Lesions [n]	1.505	1.04 - 1.97	**0.0000**
White Matter Lesions [n]	1.388	0.88 - 1.9	0.0000
Cerv. Spinal Cord V. [ml]	-0.694	-1.26 - -0.13	0.0220
MTR Cortex	-1.022	-1.66 - -0.38	0.0041
MTR WML	-1.207	-1.81 - -0.6	0.0005
NAWM MTR Gradient	1.002	0.39 - 1.61	0.0030
Brain Parenchymal Fraction	-1.032	-1.59 - -0.47	0.0010
Grey Matter Fraction	-0.914	-1.41 - -0.42	0.0010
Thalamus V. [ml]	-0.978	-1.59 - -0.37	0.0035
**RRMS vs. SPMS:**			
Cortical Lesions [n]	1.391	0.9 - 1.88	**0.0000**
White Matter Lesions [n]	0.709	0.1 - 1.32	**0.0286**
Cerv. Spinal Cord V. [ml]	-0.576	-1.12 - -0.04	**0.0434**
MTR Cortex	-0.563	-1.15 - 0.02	0.0685
MTR WML	-0.749	-1.33 - -0.16	**0.0165**
NAWM MTR Gradient	0.500	-0.14 - 1.14	0.1331
Brain Parenchymal Fraction	-0.724	-1.31 - -0.14	**0.0209**
Grey Matter Fraction	-0.834	-1.35 - -0.31	**0.0032**
Thalamus V. [ml]	-0.067	-0.67 - 0.53	0.8284
