# Supplementary material for: Cortical involvement determines impairment 30 years after a clinically isolated syndrome
Source: Brain. 2021 Apr 21;144(5):1384–95. doi: 10.1093/brain/awab033 (PMC8219364; doi:10.1093/brain/awab033)
Supplement: awab033_Supplementary_Data [file awab033_supplementary_data.zip › awab033_suppl-data/brain-2020-01742-File010.pdf]

CIS vs. RRMS	Estimate (Beta)	Beta 95%	P-value <
Cortical Lesions [n]	0.441	0.44 - 0.44	0.1500
White Matter Lesions [n]	1.114	0.63 - 1.6	**0.0000**
Cerv. Spinal Cord V. [ml]	-0.136	-0.73 - 0.46	0.6570
MTR Cortex	-0.789	-1.32 - -0.25	**0.0060**
MTR WML	-1.015	-1.52 - -0.51	**0.0000**
NAWM MTR Gradient	0.879	0.31 - 1.45	**0.0040**
Brain Parenchymal Fraction	-0.727	-1.19- -0.26	**0.0040**
Grey Matter Fraction	-0.199	-0.6 -0.2	0.3340
Thalamus V. [ml]	-0.908	-1.43- -0.39	**0.0000**
**CIS vs. SPMS**			
Cortical Lesions [n]	1.505	1.04 - 1.97	**0.0000**
White Matter Lesions [n]	1.388	0.88 - 1.9	**0.0000**
Cerv. Spinal Cord V. [ml]	-0.694	-1.26 - -0.13	**0.0221**
MTR Cortex	-1.022	-1.66 - -0.38	**0.0042**
MTR WML	-1.207	-1.81 - -0.6	**0.0006**
NAWM MTR Gradient	1.002	0.39 -1.61	**0.0031**
Brain Parenchymal Fraction	-1.032	-1.59 - -0.47	**0.0010**
Grey Matter Fraction	-0.914	-1.41 - -0.42	**0.0010**
Thalamus V. [ml]	-0.978	-1.59 - -0.37	**0.0036**
**RRMS vs. SPMS**			
Cortical Lesions [n]	1.391	0.9 - 1.88	**0.0000**
White Matter Lesions [n]	0.709	0.1-1.32	**0.0287**
Cerv. Spinal Cord V. [ml]	-0.576	-1.12--0.04	**0.0435**
MTR Cortex	-0.563	-1.15 -0.02	0.0686
MTR WML	-0.749	-1.33 - -0.16	**0.0165**
NAWM MTR Gradient	0.5	-0.14 - 1.14	0.1332
Brain Parenchymal Fraction	-0.724	-1.31 - -0.14	**0.0209**
Grey Matter Fraction	-0.834	-1.35 - -0.31	**0.0033**
Thalamus V. [ml]	-0.067	-0.67 -0.53	0.8284
